# Ancient Artworks and Crocus Genetics Both Support Saffron’s Origin in Early Greece

**DOI:** 10.3389/fpls.2022.834416

**Published:** 2022-02-25

**Authors:** Seyyedeh-Sanam Kazemi-Shahandashti, Ludwig Mann, Abdullah El-nagish, Dörte Harpke, Zahra Nemati, Björn Usadel, Tony Heitkam

**Affiliations:** ^1^Institute of Biological Data Science, Heinrich-Heine Universität Düsseldorf, Düsseldorf, Germany; ^2^IBG-4 Bioinformatics, Forschungszentrum Jülich, Jülich, Germany; ^3^Faculty of Biology, Institute of Botany, Technische Universität Dresden, Dresden, Germany; ^4^Botany and Microbiology Department, Faculty of Science, Sohag University, Sohag, Egypt; ^5^Leibniz Institute of Plant Genetics and Crop Plant Research (IPK), Gatersleben, Germany

**Keywords:** saffron, *Crocus sativus*, #ArtGenetics, Genome, cytogenetics, historical art, Minoan frescoes

## Abstract

Saffron crocus (*Crocus sativus*) is a male-sterile, triploid flower crop, and source of the spice and colorant saffron. For over three millennia, it was cultivated across the Mediterranean, including ancient Greece, Persia, and other cultures, later spreading all over the world. Despite saffron crocus’ early omnipresence, its origin has been the matter of a century-old debate, in terms of area and time as well as parental species contribution. While remnants of the ancient arts, crafts, and texts still provide hints on its origin, modern genetics has the potential to efficiently follow these leads, thus shedding light on new possible lines of descent. In this review, we follow ancient arts and recent genetics to trace the evolutionary origin of saffron crocus. We focus on the place and time of saffron domestication and cultivation, and address its presumed autopolyploid origin involving cytotypes of wild *Crocus cartwrightianus*. Both ancient arts from Greece, Iran, and Mesopotamia as well as recent cytogenetic and comparative next-generation sequencing approaches point to saffron’s emergence and domestication in ancient Greece, showing how both disciplines converge in tracing its origin.

## Introduction

From Greece to Iran, from paintings and dyes to spice and perfumes, saffron’s flavor and sparkly yellow color has made its trail in human history. As saffron crocus (*Crocus sativus* L.) can be traced in artworks across history, its appearance in various arts and crafts gives insights into saffron’s early geographic distribution and finally into its origin.

The Mediterranean is considered as the emergence site of many diploid *Crocus* species (Maw, 1886; Mathew, 1982), with Greece and Turkey possessing the highest number of *Crocus* taxa. Investigating ancient texts and artworks in these regions can help finding more information on early saffron domestication.

Regarding ancient texts, the first use of the word “saffron” dates back to the 12th century to the old French term *safran* that consecutively originated from the Latin *safranum*, the Arabic *za’farān*, and the Persian *zarparan* with the meaning “gold strung” (Asbaghi, 1988). As folk taxonomies were used to differentiate plant species prior to the formalization of the Linnaean system, different species of purple, autumn-flowering crocuses were not distinguished before the 18th century (Day, 2011a). Hence, at the time, most crocuses were considered as one and we cannot rely on texts for clear species information. Nevertheless, Caiola and Canini (2010) suggested that *Crocus cartwrightianus*, *Crocus thomasii*, or *Crocus hadriaticus* were the most likely species that were cited by ancient historians.

Regarding ancient arts, images can be categorized into two types: On one hand, those that are painted using pigments derived from crocus and on the other hand, those that depict saffron crocus flowers. The first type of image can give us information about the probable timeline of saffron use, whereas the second may give us information about its origin as it provides access to the morphological aspects of the crocus species used at the time. The major difference here is that one type provides scientific evidence for the time scale, whereas the other type leaves room for interpretation and speculation. Hence, the visual analysis depends on multiple aspects like placing the art in the socio-historical context, knowledge on artistic techniques as well as possible restoration approaches.

**Figure 1:**
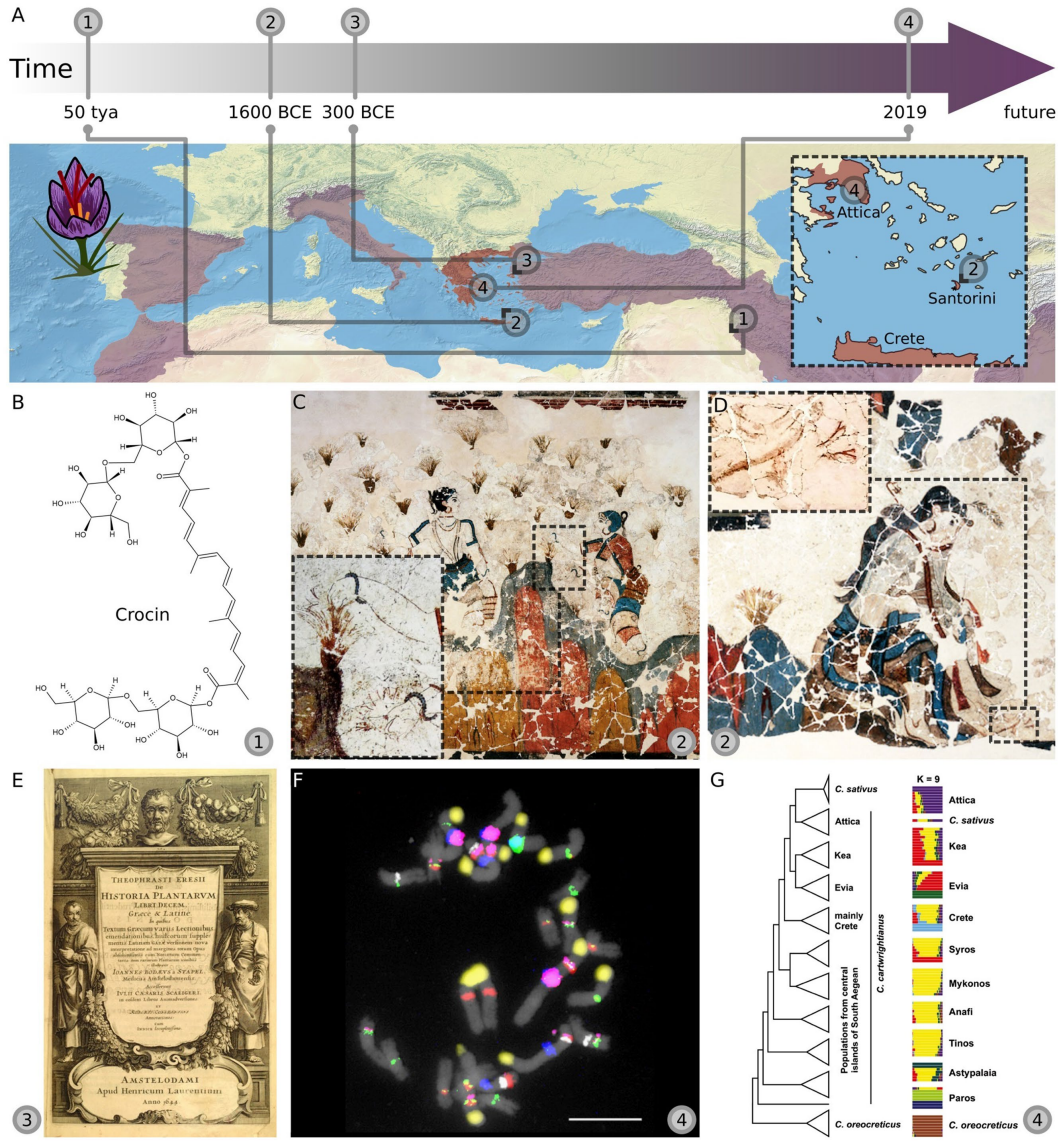
Cultivation of saffron (*Crocus sativus* L.) as traced by historic arts and modern genetics. **(A)** The timeline and map show four exemplary time-points (1–4) and their corresponding geographic locations. **(B)** The earliest evidence of “historic saffron” (any crocus species with at least partially similar traits as today’s cultivated saffron and used by humans for this particular trait) use was found in pigments from cave wall paintings. These contained crocin (structure shown) that was derived from *Crocus* flowers. These cave paintings were found in today’s north western Iraq and were created about 50,000 years ago (tya). **(C,D)** The first signs of saffron cultivation were found in Aegean Bronze Age frescoes from Crete and Santorini, dating back to 1700 to 1500 before the common era (BCE). Shown here is a part of “The Saffron Gatherers” **(C)** and “Adorants” frescos **(D)**, both found in Xeste 3 (Akrotiri, Santorini) from around 1600 BCE. **(E)** The first mentioning of saffron crocus propagation through corms was documented in *Historia Plantarum* by Theophrastus around 300 BCE. Shown here is the front cover of the Latin translation (1644). **(F,G)** Recent studies investigating the origin of saffron crocus using modern genetic approaches [multicolor FISH **(F)** and genotyping-by-sequencing GBS **(G)**] also point to early Greece as the place of origin, with Attica as the most likely region of origin. Countries that are major saffron-producers today are colored in red and purple on the map (Rukšāns, 2017). [*Image sources:* **(A)** The map was generated with Natural Earth (https://www.naturalearthdata.com/); **(B)** Crocin structure was made with ChemSketch; **(C,D)** “The Saffron Gatherers” and “Adorants” pictures from Doumas (1992) p. 152/Figure 116, p. 136/Figure 100 (contrast and brightness enhanced, layered); **(E)** *Historia Plantarum* belongs to the public domain, image provided by Andrew Dalby (Wiki commons); **(F)** FISH image reused from Schmidt et al. (2019), Figure 2Q with permission from John Wiley and Sons; **(G)** Phylogenetic tree and cluster analysis reused from Nemati et al. (2019) under CC-BY]. The timeline is not to scale.

While the use of crocus-based pigments can be traced back about 50,000 years ago to prehistoric cave paintings in northwestern Iran (today’s Iraq; Humphries, 1996; Figures 1A,B), early signs of cultivation and domestication were found later, at about 1700 before the common era (BCE), during the time of the Minoan civilization in Crete (Deo, 2003; Dewan, 2015). As saffron’s high medicinal value and antioxidant ability were recognized, its commercial value as a spice increased over the next eras, leading to its spread across the Mediterranean (Abrishami, 2004; Caiola and Canini, 2010).

Although saffron is in high demand as spice and colorant these days, its cultivation still relies on traditional knowledge and has remained largely untouched by industrialization. This is rooted in saffron’s genetic constitution as a male-sterile triploid, which can only be propagated vegetatively. Currently, any alteration in saffron production also requires change in its vegetative propagation and quality control (Caiola and Canini, 2010). In contrast, sexual propagation of saffron would result in new, desirable traits, but would involve the selection of parental genotypes. To inform the required selection process, knowledge of saffron’s genetic and geographic origin is crucial. To fill this gap, over the last decade, several studies have attempted to resolve saffron’s domestication and to clarify its auto- or allopolyploid origin.

Here, we illustrate how the insights gained from ancient artworks add to our knowledge of saffron’s origin, how they compare with the recent genetic approaches, and how they can steer further research on saffron evolution, domestication, and breeding. For this, we build on our previous work (Schmidt et al., 2019), in which we have used cytogenetics to derive the genetic origin of saffron from cytotypes of *C. cartwrightianus*, thereby excluding other *Crocus* species as parental contributors to its triploidy. In the related paper of Nemati et al. (2019), genotyping-by-sequencing (GBS) has been used to independently reach the same conclusion and to trace the genetic origin of saffron’s triploidy back to Greece. Now, we have combined forces to put the results of saffron’s genetic origin into the broader context with ancient arts. Especially as no ancient saffron samples have been uncovered from the Aegean time—that may potentially harbor aDNA—the ancient artworks are currently the only waypoints that may guide modern genomics efforts to fill in the remaining holes in saffron’s history: We go through time and explain how and where saffron use (and the use of other *Crocus* species) was described or depicted and compare the results with current genomics efforts. We put on a wider lens and draw a comprehensive picture of the current view on saffron’s history, as well as lay out what gaps are still open to be addressed in the future.

## Early Evidence of Saffron’s Origin Stems From Ancient Artworks

Evidence for the use and cultivation of saffron have been found across different cultures and times. Here, we will follow saffron’s traces across ancient artworks in a chronological manner.

The oldest evidence of crocus usage through mankind was detected in a 50,000-year-old depiction of beasts in a cave in today’s Iraq, where saffron-related pigments (including crocin) have been used (Caiola and Canini, 2010). Wild-grown “historic saffron” (“historic saffron”: any crocus species with at least partially similar traits as today’s cultivated saffron and used by humans for this particular trait) was reported to have been used by Sumerians (ca. 4100–1750 BCE) as part of their remedies and medical potions (Willard, 2002). Also, royals of old Assyria and Babylonia (2nd millennium BCE) used “historic saffron” as a treatment for several diseases and had their courts filled with textiles dyed with “historic saffron” or later possibly cultivated saffron, which were supplied by Phoenician traders (Basker and Negbi, 1983; Mousavi and Bathaie, 2011; Dewan, 2015).

The most prominent pictures of potentially cultivated saffron in historic artworks were created during the Aegean Bronze Age, particularly influenced by the Minoan civilization, dating back to 1700–1500 BCE (Deo, 2003; Dewan, 2015). Important excavation sites are located on the islands of Crete and Thera (today’s Santorini) both belonging to today’s Greece. For this review, we will mainly focus on the crocus depictions in ancient frescoes, but will also address ceramics and cloth found in Akrotiri (Thera) and Knossos (Crete; Warren, 2005).

The most relevant representations for our review show the gathering of saffron (Figures 1C,D) and its usage in cultural rituals on multiple frescoes. These were found in remains of historical buildings (“Xeste 3,” “West House,” and others) in Akrotiri. The oldest saffron-depicting fresco (“The Saffron Gatherers,” sometimes referred to as “Blue Monkeys/Boys” fresco); however, was found in the so-called House of Frescoes in Knossos and was dated to approximately 1700 BCE. With the amount and prominence of saffron flowers depicted, these frescoes clearly demonstrate the importance of saffron around 1700–1500 BCE in the Aegean Bronze Age (Dewan, 2015).

The crocuses depicted on the frescoes in Xeste 3 show distinctive similarities with modern cultivated saffron (*C. sativus*) such as their long and intensively red stigmas overtopping the dark violet petals. These visual characteristics are also found on other artistic representations of crocus flowers on ceramics and cloth (Day, 2011b; Dewan, 2015; Marinatos, 2015). Typical for this time was the syllabic script Linear B (Shelmerdine, 1998). The ideogram (script character) representing saffron shows very long stigmas overtopping three pictured petals (Day, 2011a; Dewan, 2015) matching all afore-mentioned representations as well as today’s cultivated saffron. This leads to the assumption that most likely all depicted phenotypes across Akrotiri and other excavation sites belong to only a single *Crocus* species. It is particularly striking that these representations seem to show remarkable differences compared to most of the *Crocus* species natively occurring on Crete and the surrounding islands today, such as *Crocus boryi*, *Crocus oreocreticus*, *Crocus sieberi*, *Crocus tournefortii*, *Crocus laevigatus* (Greece mainland and Santorini), *Crocus ruksansii* (Karpathos), *Crocus rhodensis* (Rhodes), *Crocus mazziaricus*, *Crocus olivieri*, and *Crocus pallasii* (Greece to Turkey; Rukšāns, 2017). The only exception here is *C. cartwrightianus* (Crete to Greece mainland), which itself shows a huge variety of differing phenotypes and is also sometimes referred to as wild saffron (Larsen et al., 2015; Rukšāns, 2017; Nemati et al., 2019; Schmidt et al., 2019). Some of the *C. cartwrightianus* phenotypes found today look very similar to those depicted in the ancient frescoes. Hence, archeologists and (paleo-)botanists classified the flowers depicted in the frescoes as either *C. sativus* or *C. cartwrightianus* (Möbius, 1933; Warren, 2000; Day, 2011a).

No remains of bulbs or other parts of the actual plants have been found in the various excavation sites making it impossible to verify which species was depicted on the artistic works (Day, 2013). The chances to find any remains in the future seem very low considering that volcanic activities (“Minoan eruption”) destroyed major parts of the island Thera (Friedrich, 2009). During this geographical event also the surrounding islands including Crete have been influenced by earthquakes and tsunamis (Friedrich, 2009).

The importance of crocus use as highlighted on the frescoes in Xeste 3 allow for some speculation: The depicted crocus plants are often planted in clumps that could be interpreted as a sort of cultivation (Morgan, 1988; Figure 1C: “The Saffron Gatherers”). Moreover, the previously mentioned Linear B tablets also include information on large amounts of traded saffron, with many dedicated fabric-processing tools found in Akrotiri (Day, 2011a). Additionally, its theorized, cosmetic use for the coloring of lips and ears during festivities was also depicted in the “Young Priestess” fresco (West House, Akrotiri; Ferrence and Bendersky, 2004; Day, 2011a; Dewan, 2015). This suggests that saffron was economically important, already forming a specialized branch of industry. Likely, this also implies that saffron collection from the wild was insufficient to meet the high demands for saffron as spice, dye, and cosmetic product, but that saffron was instead cultivated in gardens or fields.

One of the frescoes in Xeste 3 termed the “Adorants” fresco (Figure 1D) clearly shows a crocus flower harboring all typical saffron crocus traits with the bright orange/red stigmas highlighted. The woman sitting has a cut in her foot with a saffron flower depicted below. There are two interpretations on the meaning of this fresco discussed in the literature. One is that the blood colors the saffron’s stigmas red which might be seen as a sign of reaching female adulthood including menstruation (Rehak, 2004; Dewan, 2015). Others argue that the women is a goddess and the flower grows from her wounded foot (Marinatos, 2015). While both is possible, we argue that this could indeed symbolize the human-led saffron domestication efforts in the Aegean Bronze Age. In the later Minoan ages, frescoes of saffron became less common, but it continued to be depicted on remains found of all kinds of ceramics and clothing (Ferrence and Bendersky, 2004; Day, 2011a; Dewan, 2015).

Despite the most reliable evidence of the cultivation of saffron being found in the Aegean Bronze Age culture, saffron artworks have also been found around the same time in other countries, such as Egypt and Persia. In ancient Egypt, Theban tombs of five high officials whose career spanned the time from Queen Hatshepsut through the early reign of Amenophis III (New Kingdom; c. 1480–1380 BCE) depict men carrying textiles to be presented as a tribute. Style of ceilings and wall-paintings in these tombs is Aegean and reflects artistic motifs derived from saffron-colored textiles (Wachsmann and Wachsmann 1987; Barber 1991; Dewan 2015). The individuals appearing in these paintings are dressed in a stylized version of Aegean Bronze Age costumes that resemble those in the Procession Fresco from Knossos (Rehak 1996). The adjacent hieroglyphic inscriptions which appear above those men geographically identifies them as Aegean natives, the Keftiu, which is the Egyptian name for Crete, and describes them as people living in the “Islands in the midst of the sea” (Wachsmann and Wachsmann, 1987; Barber, 1991; Panagiotopoulos, 2002; Matić, 2019). Although no record of saffron cultivation by Ancient Egyptians can be found, detailed historic information of saffron usage for several medical purposes is mentioned in the Papyrus Ebers (1550 BCE; Toellner, 2000; Mousavi and Bathaie, 2011). Moreover, the rareness of saffron-dyed Minoan textiles in Egypt reflects why they were considered ideal as elite gifts to Egyptian royals and support the notion that saffron products was mainly imported from Crete which was home to a thriving textile industry (Feldman, 2002; Mousavi and Bathaie, 2011; Dewan, 2015).

Historic data indicate that Persian “historic saffron” (possibly *C. haussknechtii*) was cultivated in Derbena, Khorasan by the 10th century BCE and also during the Median Kingdom (708–550 BCE) near Zagros and Alvand mountains, suggesting that ancient Persians were one of the early nations who cultivated saffron instead of gathering wild crocus flowers (Dadkhah et al., 2003; Caiola and Canini, 2010; Mousavi and Bathaie, 2011). The main applications of saffron in Persian art were as a dye in royal carpets and funeral shrouds as well as in paper colorings and Persian miniature paintings to prevent the corrosive effect of the verdigris pigments (Willard, 2002; Barkeshli, 2016; Dehboneh et al., 2019).

Despite these many occurrences across artworks of the ancient world, the first written evidence of cultivated saffron (*C. sativus*) is only found around 350–300 BCE in the *Historia Plantarum* (Theophrastus, n.d.; Figure 1E), where the saffron-specific propagation through corms is described in detail. Hence, understanding and interpreting the ancient arts can push this time limit back for over a millennium, assisting us in formulating theories, and enabling modern saffron genetics to follow and verify these leads.

## Genetics Pinpoints the Origin of Saffron Crocus to Ancient Greece

Although saffron’s origin has been under debate for over a century, with a large body of work attempting to clarify its genetic and geographic origin (reviewed in Koocheki and Khajeh-Hosseini, 2020), the history of saffron domestication remained unresolved until recently. Instead of chromosome pairs, saffron has eight chromosome triplets with a chromosome configuration of 2n = 3x = 24 and a triploid genome size of 10.5 Gb (Chichiriccò, 1984; Brandizzi and Caiola, 1998; Agayev, 2002; Schmidt et al., 2019). Saffron’s triploidy is the cause for many genetic peculiarities of saffron.

### Saffron Is Infertile and Can Only Be Propagated Vegetatively

The triploidy of saffron crocus results in a disturbed meiosis leading to at least partial sterility. As result of erroneous chromosome pairing, meiosis progresses only incompletely and yields abnormal pollen (Chichiriccò, 1984; Rashed-Mohassel, 2020). In consequence, cross-fertilization between *C. sativus* and other species is limited (Caiola, 1999; Caiola et al., 2000). Nevertheless, as the *C. sativus* pollen tube is incapable to penetrate the *C. sativus* ovule, a self-incompatible species was suggested as the most likely progenitor (Chichiriccò and Caiola, 1986). Although pollen infertility is higher than the ovule sterility (Chichiriccò, 1984), irregular chromosome arrays also occur in megaspores, making them genetically unbalanced and infertile (Caiola and Canini, 2010). Along the same lines, haploid gametes with a whole set of chromosomes only infrequently form in triploids; another cause of saffron’s sterility. A genetic regulation of chromosome segregation as was observed for hexaploid bread wheat (Riley and Chapman, 1958; Sears, 1976; Koo et al., 2017) or postulated for pentaploid dogroses (Täckholm, 1920; Herklotz and Ritz, 2017) is either absent or yet undiscovered. Similarly, there have been no reports of hexaploid saffron that may have emerged from triploid saffron as it was occasionally observed for other triploid plant species that used hexaploidization as a route to regain fertility (Husband, 2004). Therefore, the vegetative propagation by daughter corms is considered as the only way of saffron reproduction (Fernández, 2004; Gresta et al., 2008; Nehvi et al., 2010). Nevertheless, the multiplication rate of daughter corms reduces saffron productivity, rendering high-quality propagation material crucially important (Renau-Morata et al., 2013).

### Absence of Genetic Variation Among Saffron Accessions Suggests That Saffron Only Originated Once

As saffron is effectively sterile, it cannot generate genetic variation through recombination during sexual reproduction (Fernández, 2004). Intriguingly, phenotypic differences are still accumulating in today’s saffron accessions. After clonal selection, Agayev et al. (2009) could for instance select a population of corms that was characterized by very different phenotypes. The majority of studies only detected insignificant amounts of genetic variability among saffron accessions, if at all, using molecular marker technologies, such as simple sequence repeats (SSRs), EST-derived SSRs, and amplified fragment length polymorphisms (AFLPs; Fluch et al., 2010; Siracusa et al., 2013). Hence, it is generally accepted that triploid saffron crocus emerged only once and was then distributed across the world (Rubio-Moraga et al., 2009; Fluch et al., 2010; Siracusa et al., 2013; Babaei et al., 2014; Alsayied et al., 2015; Nemati et al., 2019). However, a recent study by Busconi et al. (2021) reported a surprising amount of genetic differences (single-nucleotide polymorphisms, SNPs). This was detected by a genome-wide approach, in which primarily the global DNA methylation across five different saffron accessions was analyzed and a high epigenetic variability has been shown. These findings indicate that saffron has a higher genetic variability than previously assumed or detected. Moreover, the study confirmed the previously suggested epigenetic variation within accessions according to their geographic origin. Using canonical and methylation-sensitive AFLP (MS-AFLP) markers, 112 accessions from the World Saffron and Crocus Collection were compared before, yielding only low genetic but high epigenetic variability (Busconi et al., 2015). After prolonged co-cultivation of different saffron accessions in the same environment, their DNA methylation profiles converged, suggesting that saffron epi-genotypes are a result of adaptation to the environment (Busconi et al., 2018). This suggests that epigenetics, especially DNA methylation are likely the main cause for phenotypic variability within saffron accessions with genetic modification playing a yet not fully resolved role. A major key point to overcome the current lack of knowledge will be the establishment of a reference genome sequence.

### The Debated Origin of Saffron Crocus

**Figure 2:**
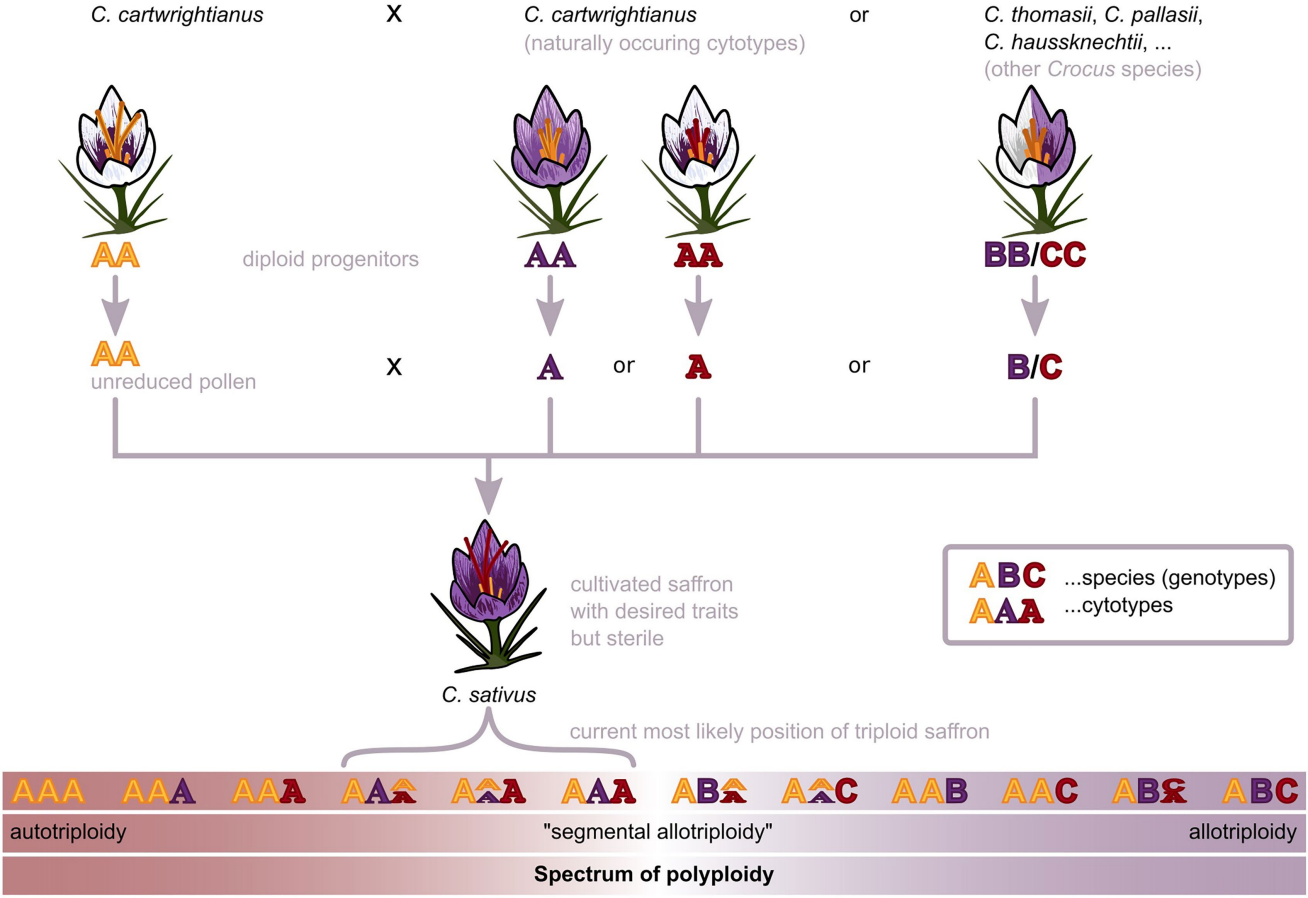
Scenarios for the auto- or allotriploid origin of saffron crocus (*Crocus sativus* L.). Saffron crocus is triploid and sterile and, hence, can only be propagated vegetatively through corms. How this triploidy emerged has been debated for over a century, with two main hypotheses being brought forward: On the one hand, saffron may have originated with genome contributions from a single species, likely *Crocus carthwrightianus* (autotriploid scenario). On the other hand, at least two species may have contributed to the genome of saffron crocus, with one likely being *C. cartwrightianus* (allotriploid scenario). Both scenarios represent extreme cases along a gradual spectrum from auto- toward allopolyploidy, as indicated by the gradual arrangement in the figure. The current position of saffron supported by several recent studies tends to be more on the autotriploid side of the spectrum of polyploidy (Nemati et al., 2019; Schmidt et al., 2019). The capital letters represent sub-genomes from different species, with different cytotypes being represented by different typefaces. The representation of the spectrum of polyploidy using letter triplets only serves as symbolic visualization and may not represent all possible scenarios.

Regarding saffron crocus’ emergence by triploidization, a major question targets the nature of its triploidy. Various types of polyploidy are commonly observed in plants, including, for example, allopolyploids, segmental allopolyploids, and autopolyploids (Figure 2). Whereas allopolyploidy (at least two parental species contribute to the polyploid state) and autopolyploidy (only one parental species contributes to the polyploid state) denote extreme cases along a gradual spectrum, segmental allopolyploids are considered as an intermediate form between true autopolyploidy and allopolyploidy (Stebbins, 1947, 1950; Sattler et al., 2016; Mason and Wendel, 2020). For saffron crocus, two main scenarios were put forward to explain the origin of saffron’s triploidy (see also Figure 2):

- Allotriploidy, originating by hybridization between individuals of different species (Frello and Heslop-Harrison, 2000; Alavi-Kia et al., 2008; Petersen et al., 2008; Tsaftaris et al., 2011; Gismondi et al., 2013; Erol et al., 2014; Alsayied et al., 2015); and
- Autotriploidy, originating by hybridization between individuals of the same species (Mathew, 1999; Negbi and Negbi, 2002; Jacobsen and Ørgaard, 2004; Nemati et al., 2019; Schmidt et al., 2019).

Regardless of the scenario, at least one parent is generally assumed to be *C. cartwrightianus*, a species occurring in southeastern mainland Greece and on Aegean islands. *Crocus cartwrightianus*, also called wild saffron, often has shorter and lighter stigmas with a less prominent aroma (Mathew, 1999; Negbi and Negbi, 2002; Jacobsen and Ørgaard, 2004). Its stigmas have served as a “historic saffron” source even before the domestication and trait enhancements of *C. sativus*. The close relationship between *C. sativus* and *C. cartwrightianus* has been confirmed by multiple observations, such as their similar phenotypes (Mathew, 1977; Caiola et al., 2010), similar pigment composition (Nørbæk et al., 2002), as well as similar genome size and base composition (Brandizzi and Caiola, 1998; Schmidt et al., 2019). Due to these many similarities, it was already suggested four decades ago that *C. cartwrightianus* is one of the most likely progenitors of saffron crocus (Mathew, 1982). However, the contribution of additional species remained under debate.

Favoring allotriploidy (scenario I), Caiola (1999) suggested the out-breeding of crocus diploids, as the origin of *C. sativus*. *C. cartwrightianus*, and *C. thomasii* were then introduced as the most probable ancestors (Brighton, 1977; Mathew, 1999). Intriguingly, Agayev (2002) and Agayev et al. (2010) showed that the triplet 5 in *C. sativus* contains heteromorphic chromosomes, already an indicator that at least two different genotypes have been involved in saffron’s domestication history. Considering allotriploidy as the origin of saffron prompted scientists to focus on its probable progenitors: Most consider *C. cartwrightianus* as the most probable donor of the diploid genome and either *C. thomasii*, *C. pallasii*, or *C. haussknechtii*, as possible second parent (Frello and Heslop-Harrison, 2000; Alavi-Kia et al., 2008; Petersen et al., 2008; Tsaftaris et al., 2011; Gismondi et al., 2013; Erol et al., 2014; Alsayied et al., 2015).

Contrary to this hypothesis, the autotriploidy (scenario II) of saffron crocus is also supported by a range of observations: Especially the high similarity between *C. sativus* and *C. cartwrightianus* suggests that *C. cartwrightianus* was the sole saffron progenitor, contributing all three chromosome sets (Mathew, 1999; Negbi and Negbi, 2002; Jacobsen and Ørgaard, 2004). In 2019, two complementary studies added evidence that supported saffron’s autotriploid origin: Schmidt et al. (2019) used a multi-color cytogenetics approach to identify *C. sativus* as a hybrid of heterogeneous *C. cartwrightianus* cytotypes (Figure 1F). This was independently verified by Nemati et al. (2019), whose GBS experiments suggested that saffron crocus evolved in Attica as a hybrid of two different genotypes of *C. cartwrightianus* (Figure 1G). Both studies concluded that the natural variation among different cyto- and genotypes of *C. cartwrightianus* was sufficient to explain the high level of heterozygosity within *C. sativus* (Figure 2). Over 99% of saffron’s GBS alleles were found in *C. cartwrightianus*, with almost 98% present in accessions found in the region of Attica (Nemati et al., 2019). However, as allele composition is constantly reorganized by genetic recombination, it is unlikely to identify the exact allele combination characteristic for triploid *C. sativus* (Nemati et al., 2019). Concluding, the most recent genetic studies pinpoint the origin of cultivated saffron to the region of Attica (Greece).

Nevertheless, some mysteries of saffron’s past still remain: None of the studies could yet explain the origin of the heteromorphic chromosome in triplet 5. We showed before that this chromosome has a different genomic makeup and speculate that this unusual chromosome morphology has been the result of an introgression prior to saffron’s triploidization (Schmidt et al., 2019). In any case, we are just beginning to understand the genetics and epigenetics of triploid saffron crocus that underlie its sterility, clonal growth, and phenotypic variability. Especially the identification of saffron’s parentage as well as the understanding of the epigenetic impact on phenotypes may open the way toward saffron breeding.

## Combining Art and Genetics: What We Can Learn From the Arts to Inform Genetic Approaches

Saffron crocus is the source of the most expensive spice in the world but is—unlike most other cash crops—inaccessible to classical breeding approaches. Investigating saffron crocus’ depictions in ancient arts and combining it with the current genetics can lead to insights into its early cultivation and geographical origin, opening up new breeding avenues for better equipped saffron varieties.

Comparing ancient arts and modern genetics gave us symmetric results: Historians cited *C. sativus*, *C. cartwrightianus*, or *C. haussknechtii* as the most likely species that are shown in ancient arts, including crafts from various origins (Iran, Greece, Egypt, etc.). Importantly, flowers depicted in Aegean Bronze Age frescoes were classified by archeologists and (paleo-)botanists as either *C. sativus* or *C. cartwrightianus*, showing that at least one of those species occurred in the area of today’s Greece, providing early signs of cultivation and economic relevance. For example, the “Adorants” fresco from Xeste 3 depicts crocus flowers in great detail, highlighting the typical dark-red stigmas overtopping the flower (Figure 1D), a trait that is fixed in *C. sativus*, but can also occur in the more variable *C. cartwrightianus*. Together with the clumps of flowers seen in “The Saffron Gatherers” (Figure 1C) the frescos could even be interpreted as an artistic rendition of the human influence on saffron’s domestication. Meanwhile, genetic research has provided a large body of evidence for saffron’s autotriploid origin from two different, in Greece naturally occurring, cytotypes of *C. cartwrightianus* using GBS and multicolor cytogenetics (Nemati et al., 2019; Schmidt et al., 2019). Hence, despite 90% of saffron being grown in Iran (Monks, 2015; Koocheki and Khajeh-Hosseini, 2020), most evidence points to its origin in early Greece. The Aegean Bronze Age artworks point to the islands of Crete and Santorini (Thera), where the most prominent frescoes can be found. Indeed, the latest GBS analysis corroborates the art-inspired focus on the Aegean area, but allows a greater precision: This genetics study shifts our attention slightly north to the region of Attica, as its modern *C. cartwrightianus* accessions were shown to contain 98% of all saffron alleles (Nemati et al., 2019). Ideally, to follow up these leads, plant remains from the ancient Aegean Bronze Age would be needed to confirm these hypotheses—for species identification and specifically to serve as a template for comparative ancient DNA genomics. Fossil, microfossil, herbarium material or otherwise preserved plant remains have already led to the understanding of the genetic history of several crop species (van Heerwaarden et al., 2011; Scott et al., 2019). Nevertheless, so far, no ancient crocus remains were reported that geneticists could tackle.

As discussed above, the phenotypic differences among saffron accessions likely originate from mostly epigenetic variability and do not point to multiple independent origins. In the future, comparative epigenomics will give a better understanding of saffron’s genetic and epigenetic composition, and offer new insights on its parental species contribution. Nevertheless, the generation of a full genome assembly will face considerable challenges, including saffron’s triploidy, the high level of genome heterozygosity, its large genome, and its high percentage of repetitive DNA. Similarly, the stability of saffron’s divergent phenotypes in consecutive growing seasons indicates that epigenetics may play a large role in phenotype breeding. Building on what we can learn from the ancient arts, further genomics approaches will likely result in clarifying the impact of early saffron domestication on its genome and also gives us a better outlook on the genetic and epigenetic basis of saffron’s quality traits that can be eventually implemented in the improvement of saffron quality and production.

## Author Contributions

S-SK-S, LM, and AE wrote the manuscript, created the figures, and contributed equally. TH initiated, outlined, and coordinated this endeavor. TH, BU, ZN, and DH guided the project intellectually and contributed to writing in their respective fields of expertise. All authors contributed to the article and approved the submitted version.

## Funding

We acknowledge funding from the Deutsche Forschungs gemeinschaft (DFG 433081887), awarded to BU (98/21-1), TH (HE 7194/2-1), and Frank Blattner (BL 462/19-1) as well as from the Egyptian Ministry of Higher Education awarded to AE (Call 2019-2020). We also thank the Open Access Funding by the Publication Fund of the TU Dresden and Sächsische Landesbibliothek – Staats- und Universitätsbibliothek (SLUB).

## Conflict of Interest

The authors declare that the research was conducted in the absence of any commercial or financial relationships that could be construed as a potential conflict of interest.

## Publisher’s Note

All claims expressed in this article are solely those of the authors and do not necessarily represent those of their affiliated organizations, or those of the publisher, the editors and the reviewers. Any product that may be evaluated in this article, or claim that may be made by its manufacturer, is not guaranteed or endorsed by the publisher.
